# Rumphellaones B and C, New 4,5-*Seco*-Caryophyllane Sesquiterpenoids from *Rumphella antipathies*

**DOI:** 10.3390/molecules190812320

**Published:** 2014-08-14

**Authors:** Hsu-Ming Chung, Wei-Hsien Wang, Tsong-Long Hwang, Jan-Jung Li, Lee-Shing Fang, Yang-Chang Wu, Ping-Jyun Sung

**Affiliations:** 1 Department of Applied Chemistry, National Pingtung University, Pingtung 900, Taiwan; E-Mail: shiuanmin@mail.npue.edu.tw; 2 National Museum of Marine Biology and Aquarium, Pingtung 944, Taiwan; E-Mails: whw@nmmba.gov.tw (W.-H.W.); jj@nmmba.gov.tw (J.-J.L.); 3 Department of Marine Biotechnology and Resources and Asia-Pacific Ocean Research Center, National Sun Yat-sen University, Kaohsiung 804, Taiwan; 4 Graduate Institute of Natural Products, Chang Gung University, Taoyuan 333, Taiwan; E-Mail: htl@mail.cgu.edu.tw; 5 Department of Sport, Health and Leisure, Cheng Shiu University, Kaohsiung 833, Taiwan; E-Mail: lsfang@csu.edu.tw; 6 School of Pharmacy, College of Pharmacy, China Medical University, Taichung 404, Taiwan; 7 Chinese Medicine Research and Development Center, China Medical University Hospital, Taichung 404, Taiwan; 8 Center for Molecular Medicine, China Medical University Hospital, Taichung 404, Taiwan; 9 Institute of Marine Biology, Department of Life Science and Institute of Biotechnology, National Dong Hwa University, Pingtung 944, Taiwan; 10 Graduate Institute of Natural Products, Kaohsiung Medical University, Kaohsiung 807, Taiwan

**Keywords:** *Rumphella antipathies*, rumphellaones B and C, caryophyllane-type sesquiterpenoid, anti-inflammatory activity

## Abstract

Two new 4,5-*seco*-caryophyllane sesquiterpenoids, rumphellaones B (**1**) and C (**2**), which were found to possess unprecedented γ-lactone moieties, were obtained from the gorgonian coral *Rumphella antipathies*. The structures of **1** and **2** were elucidated by spectroscopic methods and compound **2** was found to display modest inhibitory effects on the generation of superoxide anions and the release of elastase by human neutrophils at a concentration of 10 μg/mL.

## 1. Introduction

**Scheme 1:**
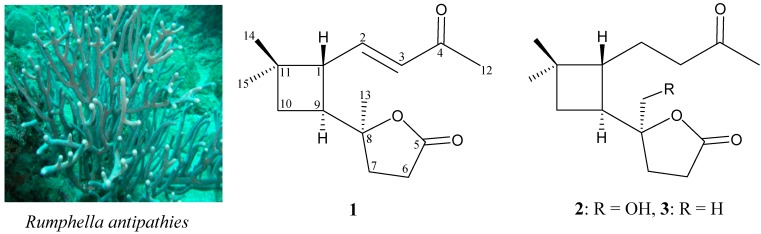
The gorgonian coral *Rumphella antipathies* and the structures of rumphellaones B (**1**), C (**2**) and A (**3**).

The chemical constituents of gorgonian corals of the genus *Rumphella*, which are widely distributed in the subtropical and tropical waters of the Indo-Pacific Ocean have been investigated for ecological and medical uses [1,2,3]. As part of our ongoing investigation into the isolation of new substances from marine invertebrates collected in the waters of Taiwan, an intersection of the Kuroshio and Oyashio currents, the chemical constituents of an organic extract of the gorgonian coral *Rumphella antipathies* (Scheme 1) which displayed meaningful signals in NMR studies were studied. Previous chemical investigations on *R. antipathies* yielded a series of caryophyllane-type sesquiterpenoid analogues, including kobusone [4], isokobusone [5], rumphellolides A–I [6,7,8,9], and rumphellatins A–D [10,11,12], which mostly possess a bicyclo[7.2.0] carbon skeleton. Moreover, in an our previous study, the first 4,5-*seco*-caryophyllane derivative, rumphellaone A [13], was isolated from *R. antipathies*. In further studies on this interesting organism, two new 4,5-*seco*-caryophyllane derivatives, rumphellaones B (**1**) and C (**2**), were isolated. In this paper, we describe the isolation, structure determination and anti-inflammatory properties of compounds **1** and **2** (Scheme 1).

Rumphella antipathies

## 2. Results and Discussion

**Figure 1:**
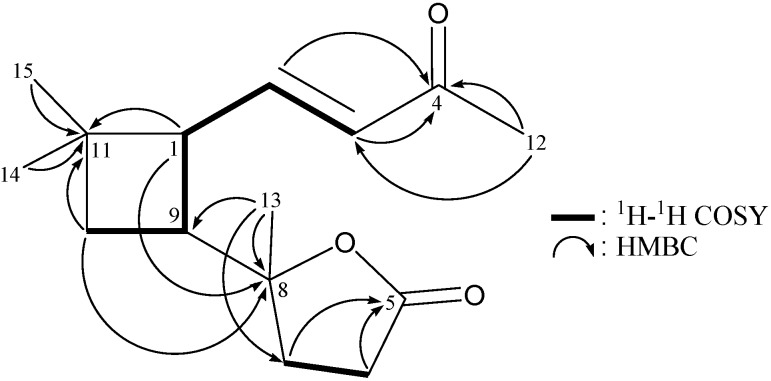
Selective key ^1^H-^1^H COSY and HMBCcorrelations for **1**.

Rumphellaone B (**1**) was isolated as a colorless oil that gave a pseudomolecular ion [M–H]^+^ at *m/z* 249.1493 in the HRESIMS, indicating the molecular formula C_15_H_22_O_3_ (calcd. for C_15_H_21_O_3_, 249.1485) implying five degrees of unsaturation. IR absorptions were observed at 1,767 and 1,712 cm^−1^, suggesting the presence of γ-lactone and α,β-unsaturated ketone groups. The ^13^C-NMR and DEPT spectra of **1** (Table 1) showed that this compound has 15 carbons, including four methyls, three sp^3^ methylenes, two sp^3^ methines, two sp^2^ methines and four quaternary carbons (including an oxygenated quaternary carbon, an ester carbonyl and a ketone carbonyl). From the ^13^C-NMR data, three degrees of unsaturation were accounted for and **1** must thus be a compound with two rings. From the ^1^H-^1^H COSY experiment of **1** (Table 1 and Figure 1), it was possible to establish the spin systems that map out the proton sequences from H_2_-10/H-9/H-1/H-2/H-3 and H_2_-6/H_2_-7, which were assembled with the assistance of an HMBC experiment (Table 1 and Figure 1). The HMBC correlations between protons and quaternary carbons of **1**, such as H-2, H-3, H_3_-12/C-4; H_2_-6, H_2_-7/C-5; H-1, H_2_-10, H_3_-13/C-8; and H-1, H_2_-10, H_3_-14, H_3_-15/C-11 permitted elucidation of the main carbon skeleton of **1**. The tertiary methyls at C-4 and C-8 were confirmed by the HMBC correlations between H_3_-12/C-3, -4 and H_3_-13/C-7, -8, -9, respectively. Moreover, two tertiary methyls at C-11 were elucidated by the HMBC correlations between H_3_-14/C-1, -10, -11, -15 and H_3_-15/C-1, -10, -11, -14. The linkage between the fragments cyclobutane and γ-lactone was established by the HMBC correlations between H-1, H_2_-10/C-8 and H_3_-13/C-9. Based on the consideration of molecular formula, an oxygen atom had to be placed between the C-5 carbonyl carbon (*δ*_C_ 176.7) and the C-8 oxygenated quaternary carbon (*δ*_C_ 86.6) to form a γ-lactone moiety.

**Table 1:** ^1^H (400 MHz, CDCl_3_) and ^13^C (100 MHz, CDCl_3_) NMR data, ^1^H-^1^H COSY and HMBC correlations for rumphellaone B (**1**).

Position	δ_H_ (*J* in Hz)	δ_C_, Multiple	^1^H–^1^H COSY	HMBC
1	2.78 dd (9.2, 8.4)	48.0, CH	H-2, H-9	C-2, -3, -8, -9, -11, -14, -15
2	6.77 dd (16.0, 8.4)	147.1, CH	H-1, H-3	C-4, -9
3	6.09 d (16.0)	131.4, CH	H-2	C-1, -4
4		198.1, C		
5		176.7, C		
6	2.51–2.68 m	29.1, CH_2_	H_2_-7	C-5
7	1.85–2.02 m	30.9, CH_2_	H_2_-6	C-5, -6
8		86.6, C		
9	2.39 m	43.0, CH	H-1, H_2_-10	C-1, -2, -10
10	1.51–1.70 m	33.1, CH_2_	H-9	C-1, -8, -9, -11, -14, -15
11		36.0, C		
12	2.25 s	27.4, CH_3_		C-3, -4
13	1.25 s	24.6, CH_3_		C-7, -8, -9
14	1.04 s	23.8, CH_3_		C-1, -10, -11, -15
15	1.10 s	29.6, CH_3_		C-1, -10, -11, -14

**Figure 2:**
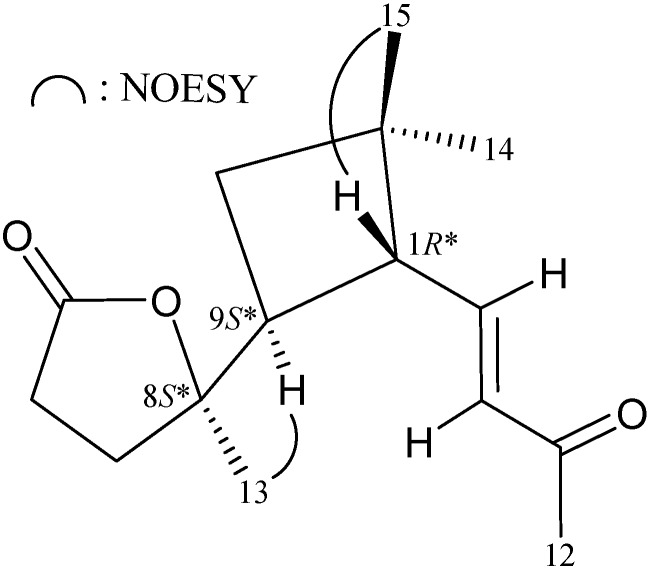
Selective key NOESY correlations for **1**.

The relative configuration of **1** was established by an analysis of interactions that were found in the NOESY experiment (Figure 2) and by vicinal ^1^H–^1^H coupling constant analysis. Due to the α-orientation of H-9, a large coupling constant was found between H-9 and H-1 (*J* = 9.2 Hz), indicating that H-1 has a β-orientation. H-1 showed a correlation with the tertiary methyl Me-15 suggesting that H-1 and H_3_-15 are located on the same face. Me-13 showed an interaction with H-9 and by comparison the NMR data of C-8 oxygenated quaternary carbon (δ_C_ 86.6) and Me-13 (δ_H_ 1.25, 3H, s; δ_C_ 24.6) with those of a similar analogue, rumphellaone A (**3**) (δ_C_ 87.2, C-8; δ_H_ 1.31, 3H, s; δ_C_ 24.9, CH_3_-13) [13], indicating that Me-13 was α-oriented at C-8. The *trans* geometry of the C-2/3 double bond was indicated by a 16.0 Hz coupling constant between H-2 (δ_H_ 6.77) and H-3 (δ_H_ 6.09). Based on the above findings, the configurations of all chiarl carbons of **1** were assigned to be 1R*, 8S* and 9S*.

Rumphellaone C (**2**) was isolated as a colorless oil that gave a pseudomolecular ion [M+Na]^+^ at *m/z* 291.1570 in the HRESIMS, indicating the molecular formula C_15_H_24_O_4_ (calcd. for C_15_H_24_O_4_Na, 291.1572) and implying four degrees of unsaturation. IR absorptions were observed at 3,435, 1,763 and 1,714 cm^−1^, suggesting the presence of hydroxy, γ-lactone and ketone groups in **2**. The ^13^C-NMR and DEPT spectra showed that this compound has 15 carbons (Table 2), including three methyls, six methylenes (including an oxymethylene), two methines and four quaternary carbons (including an oxygenated quaternary carbon and two carbonyls). Thus, from the ^13^C-NMR data, two degrees of unsaturation was accounted for, and **2** must have two rings. The ^1^H-NMR spectrum of **2** showed that all three methyl groups are isolated. In addition, six pairs of aliphatic methylene protons and two aliphatic methine protons were observed in the ^1^H-NMR spectrum of **2** (Table 2). It was found that the spectral data (IR, ^1^H and ^13^C-NMR) of **2** were similar to those of a known analogue, rumphellaone A (**3**) [13]. However, the ^1^H and ^13^C-NMR spectra revealed that the signals corresponding to the C-13 methyl group in **2** disappeared and were replaced by those of an additional hydroxymethyl group. Thus, compound **2** was found to be the 13-hydroxy derivative of **3** with the structure as described by formula **2**.

The *in vitro* anti-inflammatory effects of compounds **1** and **2** were examined and **2** displayed modestly inhibitory effects on the generation of superoxide anions (inhibition rate = 24.7%) and the release of elastase (inhibition rate = 21.1%) by human neutrophils in response to FMLP/CB at a concentration of 10 μg/mL.

**Table 2:** ^1^H (400 MHz, CDCl_3_) and ^13^C (100 MHz, CDCl_3_) NMR data, ^1^H-^1^H COSY and HMBC correlations for rumphellaone C (**2**).

Position	δ_H _(*J* in Hz)	δ_C_, Multiple	^1^H–^1^H COSY	HMBC
1	1.90 m	44.0, CH	H_2_-2, H-9	C-10, -11, -15
2	1.62 m	24.9, CH_2_	H-1, H_2_-3	C-1, -3, -4, -9, -11
3	2.36 t (7.2)	41.8, CH_2_	H_2_-2	C-1, -2, -4
4		208.6, C		
5		177.6, C		
6	2.54 ddd (18.0, 10.8, 5.2)	29.7, CH_2_	H_2_-7	C-5, -7
	2.71 ddd (18.0, 10.8, 7.6)			
7	1.94 ddd (14.2, 10.8, 7.6)	25.6, CH_2_	H_2_-6	C-5, -8, -9, -13
	2.20 ddd (14.2, 10.8, 5.2)			
8		89.5, C		
9	2.12 ddd (10.0, 10.0, 9.6)	40.2, CH	H-1, H_2_-10	n. o. ^a^
10	1.43 dd (10.4, 10.0)	33.0, CH_2_	H-9	C-1, -8, -9, -11, -14, -15
	1.57 dd (10.4, 9.6)			
11		33.5, C		
12	2.12 s	30.0, CH_3_		C-3, -4
13	3.43 d (11.6)	66.6, CH_2_		C-7, -8
	3.73 d (11.6)			
14	1.03 s	22.5, CH_3_		C-1, -10, -11, -15
15	1.07 s	30.8, CH_3_		C-1, -10, -11, -14

^a^ n. o. = not observed.

## 3. Experimental Section

### 3.1. General Experimental Procedures

Optical rotation values were measured with a Jasco P-1010 digital polarimeter (Japan Spectroscopic Corporation, Tokyo, Japan). IR spectra were obtained on a Varian Diglab FTS 1000 FT-IR spectrophotometer (Varian Inc., Palo Alto, CA, USA); peaks are reported in cm^−^^1^. NMR spectra were recorded on a Varian Mercury Plus 400 NMR spectrometer (Varian Inc.) using the residual CHCl_3_ signal (δ_H_ 7.26 ppm) as the internal standard for ^1^H-NMR and CDCl_3_ (δ_C_ 77.1 ppm) for ^13^C-NMR. Coupling constants (*J*) are given in Hz. ESIMS and HRESIMS were recorded using a Bruker 7 Tesla solariX FTMS system (Bruker, Bremen, Germany). Column chromatography was performed on silica gel (230–400 mesh, Merck, Darmstadt, Germany). TLC was carried out on precoated Kieselgel 60 F_254_ (0.25 mm, Merck); spots were visualized by spraying with 10% H_2_SO_4_ solution followed by heating. Normal-phase HPLC (NP-HPLC) was performed using a system comprised of a Hitachi L-7110 pump (Hitachi Ltd., Tokyo, Japan), a Hitachi L-7455 photodiode array detector (Hitachi Ltd.) and a Rheodyne 7725 injection port (Rheodyne LLC, Rohnert Park, CA, USA). A semi-preparative normal-phase column (Hibar 250 × 10 mm, LiChrospher Si 60, 5 μm, Merck, Darmstadt, Germany) was used for HPLC.

### 3.2. Animal Material

Specimens of the gorgonian coral *Rumphella antipathies* (Nutting) were collected by hand using scuba equipment off the coast of Pingtung, Southern Taiwan. A voucher specimen (specimen No. NMMBA-TWGC-010) was deposited in the National Museum of Marine Biology and Aquarium, Taiwan.

### 3.3. Extraction and Isolation

Sliced bodies of the gorgonian *R. antipathies* (wet weight 402 g, dry weight 144 g) are extracted with a mixture of methanol (MeOH) and dichloromethane (CH_2_Cl_2_) (1:1) at room temperature. The extract was partitioned between ethyl acetate (EtOAc) and H_2_O and the EtOAc layer was subjected to silica gel and eluted using *n*-hexane/EtOAc (stepwise, 25:1-pure EtOAc) to yield 29 fractions. Every fraction was checked using the ^1^H-NMR spectra. Fractions 21 and 27 were re-purified by normal phase HPLC (NP-HPLC) using a mixture of *n*-hexane and acetone as the mobile phase to afford **1** (5.0 mg, 3:1) and **2** (3.4 mg, 2:1), respectively.

*Rumphellaone B* (**1**): Colorless oil; 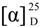 +18 (*c* 0.25, CHCl_3_); IR (neat) ν_max_ 1,767, 1,712 cm^−1^; ^1^H-NMR (CDCl_3_, 400 MHz) and ^13^C-NMR (CDCl_3_, 100 MHz) data, see Table 1; ESIMS *m/z* 249 [M − H]^+^; HRESIMS *m/z* 249.1493 (calcd. for C_15_H_2__2_O_3_–H, 249.1485).

*Rumphellaone C* (**2**): Colorless oil; 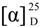 −8 (*c* 0.18, CHCl_3_); IR (neat) ν_max_ 3,435, 1,763, 1,714 cm^−1^; ^1^H-NMR(CDCl_3_, 400 MHz) and ^13^C-NMR (CDCl_3_, 100 MHz) data: see Table 2; ESIMS *m/z* 291 [M + Na]^+^; HRESIMS *m/z* 291.1570 (calcd. for C_1__5_H_24_O_4_+Na, 291.1572).

### 3.4. Generation of Superoxide Anions and Release of Elastase by Human Neutrophils

Human neutrophils were obtained by means of dextran sedimentation and Ficoll centrifugation. Measurements of superoxide anion generation and elastase release were carried out according to previously described procedures [14,15]. Briefly, superoxide anion production was assayed by monitoring the superoxide dismutase-inhibitable reduction of ferricytochrome *c*. Elastase release experiments were performed using MeO-Suc-Ala-Ala-Pro-Valp-nitroanilide as the elastase substrate. In the *in vitro* anti-inflammatory bioassay, the inhibitory effects on the generation of superoxide anion and the release of elastase by activated neutrophils were used as indicators. For significant activity of pure compounds, an inhibition rate ≥ 50% is required (inhibition rate ≤ 10%, not active; 20% ≥ inhibition rate ≥ 10%, weakly anti-inflammatory; 50% ≥ inhibition rate ≥ 20%, modestly anti-inflammatory).

## 4. Conclusions

The gorgonian coral *R. antipathies*, collected off the waters of Taiwan, has proven to be a rich source of caryophyllane- and clovane-type sesquiterpenoids. In our continuing investigation on the chemical constituents of *R. antipathies*, two new 4,5-secocaryophyllane derivatives, rumphellaones B (**1**) and C (**2**), were isolated. It is noteworthy to mention that metabolites **1** and **2** represent the second and third 4,5-secocaryophyllane derivative containing a γ-lactone moiety, respectively, and compound **2** was found to display modestly inhibitory effects on the generation of superoxide anions and the release of elastase by human neutrophils.
